# The NF-κB Transcription Factor c-Rel Modulates Group 2 Innate Lymphoid Cell Effector Functions and Drives Allergic Airway Inflammation

**DOI:** 10.3389/fimmu.2021.664218

**Published:** 2021-11-16

**Authors:** Barbara C. Mindt, Sai Sakktee Krisna, Claudia U. Duerr, Mathieu Mancini, Lara Richer, Silvia M. Vidal, Steven Gerondakis, David Langlais, Jörg H. Fritz

**Affiliations:** ^1^ McGill University Research Centre on Complex Traits (MRCCT), Montréal, QC, Canada; ^2^ Department of Microbiology and Immunology, McGill University, Montréal, QC, Canada; ^3^ Department of Physiology, McGill University, Montréal, QC, Canada; ^4^ Charité – Universitätsmedizin Berlin, Corporate Member of Freie Universität Berlin and Humboldt-Universität zu Berlin, Department of Microbiology, Infectious Diseases and Immunology, Berlin, Germany; ^5^ Department of Human Genetics, McGill University, Montréal, QC, Canada; ^6^ Department of Pathology, McGill University, Montréal, QC, Canada; ^7^ Biomedicine Discovery Institute and Department of Biochemistry and Molecular Biology, Monash University, Clayton, VIC, Australia; ^8^ McGill University Genome Centre, Montreal, QC, Canada; ^9^ FOCiS Centre of Excellence in Translational Immunology (CETI), Montréal, QC, Canada

**Keywords:** group 2 innate lymphoid cell (ILC2), IL-33, c-Rel, type 2 immunity, airway inflammation

## Abstract

Group 2 innate lymphoid cells (ILC2s) play a key role in the initiation and orchestration of early type 2 immune responses. Upon tissue damage, ILC2s are activated by alarmins such as IL-33 and rapidly secrete large amounts of type 2 signature cytokines. ILC2 activation is governed by a network of transcriptional regulators including nuclear factor (NF)-κB family transcription factors. While it is known that activating IL-33 receptor signaling results in downstream NF-κB activation, the underlying molecular mechanisms remain elusive. Here, we found that the NF-κB subunit c-Rel is required to mount effective innate pulmonary type 2 immune responses. IL-33-mediated activation of ILC2s *in vitro* as well as *in vivo* was found to induce c-Rel mRNA and protein expression. In addition, we demonstrate that IL-33-mediated activation of ILC2s leads to nuclear translocation of c-Rel in pulmonary ILC2s. Although c-Rel was found to be a critical mediator of innate pulmonary type 2 immune responses, ILC2-intrinsic deficiency of c-Rel did not have an impact on the developmental capacity of ILC2s nor affected homeostatic numbers of lung-resident ILC2s at steady state. Moreover, we demonstrate that ILC2-intrinsic deficiency of c-Rel alters the capacity of ILC2s to upregulate the expression of ICOSL and OX40L, key stimulatory receptors, and the expression of type 2 signature cytokines IL-5, IL-9, IL-13, and granulocyte-macrophage colony-stimulating factor (GM-CSF). Collectively, our data using *Rel* ^−/−^ mice suggest that c-Rel promotes acute ILC2-driven allergic airway inflammation and suggest that c-Rel may contribute to the pathophysiology of ILC2-mediated allergic airway disease. It thereby represents a promising target for the treatment of allergic asthma, and evaluating the effect of established c-Rel inhibitors in this context would be of great clinical interest.

## Introduction

Group 2 innate lymphoid cells (ILC2s) mediate early type 2 immune responses and thereby exert key roles in the initiation and orchestration of anti-helminth immunity as well as allergic inflammation (1–5). ILC2s exhibit striking functional similarity to adaptive type 2 T helper (Th2) cells. Like Th2 cells, they depend on the transcription factor GATA3 and produce type 2 signature cytokines such as interleukin (IL)-5 and IL-13 upon activation driving eosinophil recruitment as well as goblet cell hyperplasia and mucus production, respectively (6). ILC2s are located at barrier surfaces including the lung and, contrary to Th2 cells, lack the expression of specific antigen receptors (6). They instead become activated in an antigen-independent fashion in response to environmental cues such as alarmins IL-25, IL-33, and/or thymic stromal lymphopoietin (TSLP) that are released upon tissue perturbation (6).

Among these alarmins, IL-33 has been described as the most potent activator of lung ILC2s (7). IL-33 signals through the heterodimeric IL-33 receptor (IL-33R) composed of the ligand-binding chain ST2 and IL-1 receptor accessory protein (IL-1RacP) (8). While it is known that activating IL-33R signaling results in downstream nuclear factor (NF)-κB activation, the underlying molecular mechanisms in ILC2s remain largely elusive (8). NF-κB signaling is mediated by homo- or heterodimers of proteins of the Rel/NF-κB transcription factor superfamily including NF-κB1 (p50), NF-κB2 (p52), RelA (p65), RelB, and c-Rel (9). Despite sharing a common DNA recognition motif, knockout mice lacking individual Rel/NF-κB family members exhibit non-redundant phenotypes (10, 11). Moreover, differential expression patterns in tissues and responses to receptor signals as well as target gene specificity indicate that distinct NF-κB subunits exert unique physiological roles (10, 11). While stimulation of lung ILC2s with IL-33 results in phosphorylation of RelA and treatment with a pan-NF-κB inhibitor impairs ILC2 effector functions (12–15), RelB was shown to be an intrinsic repressor of ILC2s (16). On the contrary, the function of c-Rel in ILC2s remains undefined. c-Rel has been shown to promote type 2 immune responses upon ovalbumin (OVA)-induced allergic airway inflammation (17). Furthermore, inhibition of c-Rel in a mouse model of house dust mite-mediated allergic inflammation resulted in reduced levels of IL-13 and airway hyper-reactivity as well as lung inflammation (18, 19) and inhibited eosinophil recruitment in an OVA model of chronic asthma (20). Since ILC2s are main drivers of allergic asthma, we aimed to investigate whether c-Rel exhibits similar effects during ILC2-driven allergic airway inflammation.

In the present study, we found that deficiency in c-Rel severely diminished early pulmonary ILC2-driven type 2 responses to intranasal IL-33 administration. We further demonstrate that c-Rel expression and activation in ILC2s are positively regulated by IL-33.

## Materials and Methods

### Mice

C57BL/6J wild-type (WT) mice were originally purchased from The Jackson Laboratory (Bar Harbor, ME) and bred in house. *Rel* ^−/−^ mice have been previously described and were kindly provided by Dr. Steve Gerondakis (Monash University) (21). All animals were maintained on a C57BL/6J background and were bred and housed under specific pathogen-free conditions with *ad libitum* access to food and water. Experiments were conducted with adult female age-matched mice (8–12 weeks) in accordance with the guidelines and policies of the Canadian Council on Animal Care and those of McGill University.

### *In Vivo* Stimulation

Mice were anesthetized with isoflurane followed by intranasal administration of either phosphate-buffered saline (PBS) as a control or 500 ng carrier-free recombinant murine IL-33 (rmIL-33, R&D Systems) per mouse in a total volume of 40 μl. Mice were challenged with PBS or IL-33 for three consecutive days (d0, d1, and d2), and lungs were isolated and analyzed 24 h after the last treatment.

### Lung Histopathology

Lungs were inflated with 10% buffered formalin, fixed overnight, and transferred to 70% ethanol. Fixed lungs were embedded in paraffin, sectioned, and stained with hematoxylin and eosin following standard procedures. A histologic disease score from 0 to 4 was attributed based on peribronchial, perivascular, and parenchymal immune cell infiltration.

### Preparation of Single-Cell Suspensions From Tissue

Lungs were isolated, finely minced, and digested in Roswell Park Memorial Institute (RPMI)-1640 containing 5% fetal bovine serum (FBS), 0.2 mg/ml of Liberase™ TM (Roche), and 0.1 mg/ml of DNase I (Roche) at 37°C. Digested lungs were homogenized with a 5-ml syringe attached to an 18G needle, filtered through a 70 μM cell strainer, and washed with PBS. Red blood cells were lysed using Red Blood Cell Lysing Buffer Hybri-Max™ (Sigma-Aldrich), and cells were washed with fluorescence-activated cell sorting (FACS) buffer (PBS + 2% FBS). Small intestines were isolated, opened longitudinally, cut in small pieces, and vortexed in gut buffer (Hanks’ Balanced Salt Solution (HBSS) + 2% FBS + 15 mM of HEPES) to remove fecal matter. Intestinal epithelial cells were removed by incubation in gut buffer containing 5 mM of EDTA for 20 min. Lamina propria was subsequently digested in RPMI-1640 supplemented with 5% FBS, 15 mM of HEPES, 0.1 mg/ml of Liberase™ TM (Roche), and 0.1 mg/ml of DNase I (Roche) at 37°C for 15 min. Digestion suspensions were filtered through a 70 μM cell strainer to obtain a single-cell suspension, and cells were washed with FACS buffer.

### Flow Cytometry

Pelleted single-cell suspensions were resuspended in 2.4G2 hybridoma supernatant dilution and incubated for 15 min on ice to block Fc receptors. Cells were subsequently stained with antibody dilutions prepared in PBS supplemented with 2% FBS for 30 min on ice. Dead cells were excluded by staining with Fixable Viability Dye eFluor™ 780 (eBioscience) following the manufacturer’s instructions. Intracellular staining was performed using the FoxP3/Transcription Factor Staining Buffer Set (eBioscience) according to the manufacturer’s protocol. Stained cell suspensions were acquired on a BD FACSCanto™ II System (BD Biosciences), a BD LSRFortessa™ Cell Analyzer (BD Biosciences) or sorted on a BD FACSAria™ III Cell Sorter (BD Biosciences). Flow cytometry data were analyzed using FlowJo X (BD Biosciences). All antibodies used for flow cytometry analyses are listed in **Table 1**.

**Table 1:** Antibodies.

TARGET	CLONE	SOURCE
**Flow cytometry antibodies**		
CD3ϵ	145-2C11	eBioscience
CD5	53-7.3	eBioscience
CD11b	M1/70	eBioscience
CD11c	N418	eBioscience
CD19	eBio1D3	eBioscience
CD25	PC61.5	eBioscience
CD26	H194-112	eBioscience
CD45	30-F11	BioLegend
CD45R (B220)	RA3-6B2	eBioscience
CD90.2 (Thy-1.2)	53-2.1	BioLegend
CD117 (c-Kit)	2B8	eBioscience
CD127	A7R34 or SB/199	BioLegend
CD172a (SIRPa)	P84	eBioscience
CD252 (OX40L)	RM134L	BD Biosciences
CD275 (ICOSL)	HK5.3	eBioscience
CD278 (ICOS)	7E.17G9	BD Biosciences
c-Rel	1RELAH5	eBioscience
F4/80	BM8	eBioscience
FcϵR1α	MAR-1	eBioscience
GATA3	TWAJ	eBioscience
GM-CSF	MP1-22E9	BioLegend
IL-5	TRFK5	BioLegend
IL-9	RM9A4	BioLegend
IL-13	eBio13A	eBioscience
Ki-67	SolA15	eBioscience
KLRG1	2F1	eBioscience
Integrin α4β7	DATK32	BioLegend
Ly-6A/E (Sca-1)	E13-161.7	BioLegend
Ly6C	AL-21	BD Biosciences
Ly6G/Ly6C (Gr-1)	RB6-8C5	eBioscience
Ly6G	1A8	BD Biosciences
MHC Class II (I-A/I-E)	M5/114	BD Biosciences
NK1.1	PK136	eBioscience
Siglec-F	E50-2440	BD Biosciences
IL-33R	RMST2-2	eBioscience
TER-119	TER-119	eBioscience
XCR-1	ZET	eBioscience
**Western Blot antibodies**		
c-Rel	D4Y6M	Cell Signaling Technology
GAPDH	14C10	Cell Signaling Technology
Histone H2A	D6O3A	Cell Signaling Technology
Anti-rabbit IgG, HRP-linked	polyclonal	Cell Signaling Technology

### Isolation and Expansion of Bone Marrow Group 2 Innate Lymphoid Cell Progenitors

Bone marrow ILC2 progenitors were isolated and expanded as described previously with minor modifications (22). Briefly, bone marrow from the tibias and femurs was pooled, subjected to red blood cell lysis using Red Blood Cell Lysing Buffer Hybri-Max™ (Sigma-Aldrich), and sorted as lineage (CD3ε, CD5, CD11b, CD11c, Gr1, Ly6G, CD45R (B220), NK1.1, TCRβ, TCRγδ, and Ter-119)-negative, Sca-1^+^c-kit^−^CD25^+^ cells. Isolated cells were expanded in complete ILC2 medium supplemented with recombinant murine IL-2, IL-7, IL-25, and IL-33 (all 50 ng/ml; R&D Systems) and TSLP (20 ng/ml; R&D Systems). After 2–3 weeks of expansion, cells were rested for 72 h in IL-2 and IL-7 (both 10 ng/ml), washed, and incubated in complete ILC2 medium without cytokines for 4 h before use in experiments.

### Isolation of Lung and Small Intestinal Group 2 Innate Lymphoid Cells

Single-cell suspensions from lung and small intestine were stained with respective surface antibodies and viability dye as described above and sorted on a BD FACSAria™ III Cell Sorter (BD Biosciences) as live CD45^+^Lin^−^Thy-1^+^ST2^+^CD25^+^ or live CD45^+^Lin^−^Thy-1^+^CD127^+^KLRG1^+^ cells, respectively. Isolated cells were cultured for 18 h in complete ILC2 medium containing recombinant murine IL-7 (10 ng/ml; R&D Systems), washed, and rested for 4 h in complete ILC2 medium without cytokines before being used in experiments.

### Western Blotting Analysis

Bone marrow-derived ILC2s were stimulated for the indicated time points with rmIL-33, and sub-cellular fractionation was performed as previously described (23). Briefly, pelleted cells were resuspended in 900 μl of PBS/0.1% NP-40 containing protease inhibitor and triturated with a micropipette to lyse the cell membranes. Three hundred microliters of lysate was collected (WC=whole-cell lysate). The remaining 600 μl were centrifuged at 13,000 *g* for 10 s, and supernatant was collected (C=cytoplasmic fraction). Finally, the remaining pellet, containing intact nuclei, was resuspended in 300 μl of PBS/0.1% NP-40 with protease inhibitor. All fractionated samples were probe-sonicated for 15 s at 60% amplitude. Protein was quantified using the Bio-Rad Protein Assay (#500-0006), as per manufacturer’s instructions. Prior to loading samples were denaturated in 3× Laemmli buffer containing sodium dodecyl sulfate (SDS) and 15% β-mercaptoethanol at 95°C for 5 min. 15 µg of protein for whole-cell lysate and cytoplasmic fraction samples, or 30 μl/one-tenth of the total nuclear fractionated samples, was separated on 8% polyacrylamide gels, with a 3% polyacrylamide stacking gel. Proteins were wet-transferred onto nitrocellulose membranes. For immunoblotting, membranes were probed with anti-c-Rel; anti-GAPDH or anti-H2A (all Cell Signaling Technology) in 0.1% T-TBS + 5% milk powder followed by incubation with anti-rabbit IgG-HRP (Cell Signaling Technology). The details of the antibodies are listed in **Table 1**.

### ImageStream Analysis

Isolated lung ILC2s were left for 4 h in medium without cytokines and left untreated or stimulated with rmIL-33 (10 ng/ml; R&D Systems) for the indicated time points. Cells were fixed and permeabilized using the FoxP3/Transcription factor staining kit (eBioscience) according to the manufacturer’s instructions and stained with anti-c-Rel, anti-CD25, and DAPI (NucBlue™ Fixed Cell ReadyProbes™ Reagent, Life Technologies). Samples were run on an ImageStreamX Mark II imaging flow cytometer (Amnis), and nuclear translocation of c-Rel in CD25^+^c-Rel^+^DAPI^+^ cells was analyzed using the similarity feature of the IDEAS software (Amnis). All antibodies are listed in **Table 1**.

### RNA Extraction and Quantitative Real-Time PCR

Total RNA from cultured ILC2s was extracted using the Quick-RNA MicroPrep Kit (Zymo Research) according to the manufacturer’s instructions. For preparation of total lung RNA, tissue was mechanically disrupted in a MagNA Lyser (Roche) followed by RNA extraction with TRIzol™ Reagent (Life Technologies) and clean-up using the RNeasy Mini kit (QIAGEN) according to the manufacturer’s instructions. cDNA was prepared using Oligo(dT)_12-18_ Primer (Life Technologies) and SuperScript™ III Reverse Transcriptase (Life Technologies). qRT-PCRs were performed with PowerUp™ SYBR™ Green Master Mix (Applied Biosystems) in a StepOnePlus™ Real-Time PCR System (Applied Biosystems). Transcript expression was normalized to *Hprt* expression levels and quantified using the comparative 2^−ΔΔCT^ method. Data are depicted as either relative expression or relative fold change compared to the mean of the control group. Primers used in this study were designed using the PrimerQuest Tool (Integrated DNA Technologies) and purchased from Integrated DNA Technologies. Sequence details of primers are provided in **Table 2**.

**Table 2:** qRT-PCR primers.

Gene	Forward primer (5' - 3')	Reverse primer (5' - 3')	Reference
*Ccl17*	GGAAGTTGGTGAGCTGGTATAA	GATGGCCTTCTTCACATGTTTG	Duerr et al. (22)
*Ccl22*	CTTCTTGCTGTGGCAATTCAG	TCACTAAACGTGATGGCAGAG	Duerr et al. (22)
*Hprt*	TCAGTCAACGGGGGACATAAA	GGGGCTGTACTGCTTAACCAG	Hernandez et al. (24)
*Il5*	CTCTGTTGACAAGCAATGAGACG	TCTTCAGTATGTCTAGCCCCTG	Mohapatra et al. (25)
*Il13*	GCAGCATGGTATGGAGTGT	TATCCTCTGGGTCCTGTAGATG	this study
*Rel*	GGATCAACTGGAGAAGGAAGATT	ATGGACCCGCATGAAGAATAG	this study

### Protein Quantification

IL-5, IL-9, IL-13, and granulocyte-macrophage colony-stimulating factor (GM-CSF) in tissue culture supernatants were determined using the respective mouse DuoSet ELISA kits (R&D Systems) according to the manufacturer’s instructions. Absorbance was measured using an Enspire™ 2300 Multilabel Reader (PerkinElmer).

### Intracellular Cytokine Staining

To determine intracellular cytokine production by isolated murine lung ILC2s, sorted cells in complete ILC2 medium (see above) were stimulated in 96-well round-bottom plates (15,000 cells/well) for 48 h with medium only; recombinant murine IL-2, IL-7, IL-9, and IL-33 (10 ng/ml; R&D Systems); or combinations thereof. GolgiPlug (BD Biosciences) was added for the last 6 h of culture. To analyze cytokine production following *in vivo* stimulation, lung cell suspensions (1 × 10^6^ cells/well in a 96-well plate) in complete ILC2 medium were stimulated with Cell Stimulation Cocktail (eBioscience) according to the manufacturer’s instructions in the presence of GolgiPlug (BD Biosciences). Cells were stained with respective surface antibodies and viability dye as described above, and intracellular cytokine staining was performed using the FoxP3/Transcription Factor Staining Buffer Set (eBioscience) according to the manufacturer’s protocol. Stained cells were acquired on a BD LSRFortessa™ Cell Analyzer (BD Biosciences) and analyzed using FlowJo X (BD Biosciences). All antibodies used for flow cytometry analyses are listed in **Table 1**.

### Statistical Analyses

All data were analyzed with GraphPad Prism software (GraphPad Software). p-Values below 0.05 were defined as statistically significant (*p < 0.05, **p < 0.01, ***p < 0.001). Unless otherwise indicated, figures display means ± standard deviation (SD). Experiment sample sizes (n), experiment replicate numbers, and statistical tests used are included in the respective figure legends.

## Results

### c-Rel Is a Critical Mediator of Innate Type 2 Immune Responses

**Figure 1:**
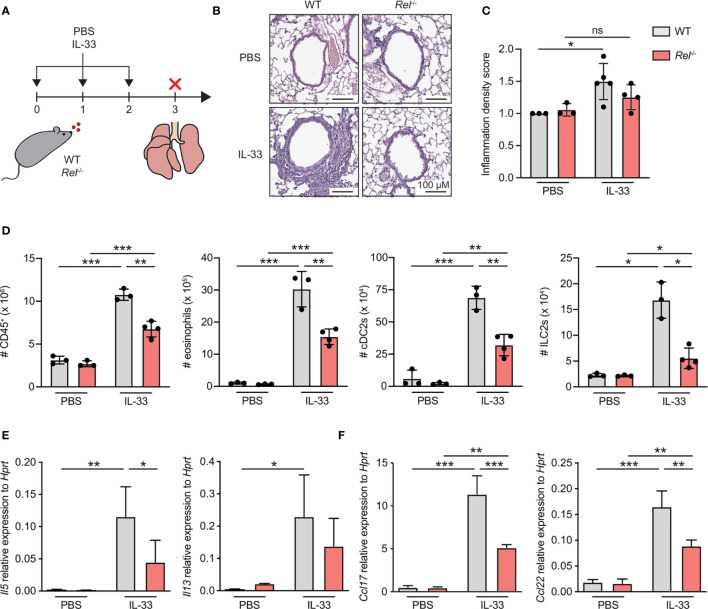
c-Rel is a critical mediator of innate type 2 immune responses. **(A)** Wild-type (WT) and c-Rel-deficient (*Rel* ^−/−^) mice were intranasally challenged for three consecutive days (day 0, 1, and 2) with phosphate-buffered saline (PBS) as a control or IL-33 (500 ng/mouse). Lungs were isolated and analyzed 24 h after the last challenge (day 3). **(B)** Microscopy of lung sections stained with hematoxylin and eosin (H&E) from WT (left panel) and *Rel* ^−/−^ mice (right panel) treated with PBS or IL-33. Scale bars, 100 µM. **(C)** Pathology score of inflammatory infiltration density assessed microscopically from H&E-stained lung sections. ns, not significant. **(D)** Total numbers of CD45^+^ leukocytes, eosinophils, type 2 conventional dendritic cells (cDC2s), and group 2 innate lymphoid cells (ILC2s) in lungs of WT (gray) or *Rel* ^−/−^ (red) mice were determined by flow cytometric analysis. Expression levels of **(E)** *Il5* and *Il13* as well as **(F)** *Ccl17* and *Ccl22* in whole lung tissue of WT (gray) and *Rel* ^−/−^ (red) animals were assessed by qRT-PCR. Data are representative of two independent experiments with n = 3–4 mice per group. Data are shown as mean ± SD with *p < 0.05, **p < 0.01, ***p < 0.001 as determined by one-way ANOVA followed by Tukey’s multiple comparisons test.

To investigate the role of c-Rel during ILC2-driven allergic airway inflammation, we challenged WT and c-Rel-deficient (*Rel* ^−/−^) mice intranasally with either PBS or IL-33 for three consecutive days and analyzed parameters of airway inflammation (**Figure 1A**). Pulmonary tissue histology showed that, when exposed to IL-33, lack of c-Rel resulted in a significant reduction in perivascular and peribronchial immune cell infiltration into lung tissue as compared with WT mice (**Figures 1B, C**). These findings could be recapitulated by flow cytometry, showing markedly decreased numbers of pulmonary CD45**^+^** leukocytes in c-Rel-deficient animals after IL-33 treatment (**Figure 1D**). Moreover, while challenge of WT mice with IL-33 resulted in a significant increase in total numbers of type 2 immunity-associated cell populations including eosinophils, type 2 dendritic cells (DC2s), and ILC2s, numbers were significantly diminished in *Rel* ^−/−^ mice (**Figure 1D** and **Supplementary Figures 1A–D**). Furthermore, c-Rel deficiency led to reduced expression of lung *Il5* transcripts with a trend towards lower *Il13* levels (**Figure 1E**). In addition, the type 2-associated chemokines *Ccl17* and *Ccl22* were greatly reduced in *Rel* ^−/−^ compared with WT mice (**Figure 1F**). In summary, compared with WT animals, c-Rel-deficient mice mount a reduced innate type 2 immune response with a significant reduction in pulmonary leukocyte infiltration, eosinophilia, DC2s, and ILC2s. Our results thus suggest that c-Rel is critical for the development of IL-33-driven allergic lung inflammation.

### c-Rel-Deficient Group 2 Innate Lymphoid Cells Exhibit No Intrinsic Defects in Their Developmental Capacity

**Figure 2:**
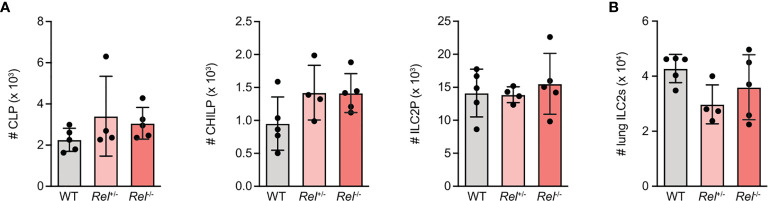
c-Rel-deficient group 2 innate lymphoid cells (ILC2s) exhibit no intrinsic defects in their developmental capacity. Flow cytometric analysis of total numbers of **(A)** ILC2 precursor populations CLP (left), CHILP, (middle) and ILC2 progenitor (ILC2P) (right) as well as **(B)** pulmonary ILC2s in wild-type (WT), heterozygous *Rel* ^+/−^, and homozygous *Rel* ^−/−^ littermate control mice. Data points are representative of two independent experiments with n = 4–5 mice per treatment group **(A, B)**. Data are shown as mean ± SD as determined by one-way ANOVA followed by Tukey’s multiple comparisons test. CLP, common lymphoid progenitor; CHILP, common helper ILC2 progenitor; ILC2P, ILC2 progenitor.

To assess whether lack of c-Rel results in ILC2-intrinsic alterations that may explain why *Rel* ^−/−^animals mount a strongly reduced innate type 2 immune response, we analyzed ILC2 progenitor numbers in the bone marrow of c-Rel sufficient (*Rel* ^+/+^), heterozygous (*Rel* ^+/−^), and c-Rel-deficient (*Rel* ^−/−^) littermate control animals. We observed that mice of all three genotypes harbored comparable numbers of common lymphoid progenitors (CLPs), as well as the more downstream common helper ILC progenitors (CHILPs), and ILC2 progenitors (ILC2Ps) (**Figure 2A** and **Supplementary Figure 2A**). In addition, there was no difference in total numbers of mature lung ILC2s (**Figure 2B**). Together, these results indicate that c-Rel is dispensable for the development of ILC2s in the bone marrow and that c-Rel deficiency does not affect homeostatic numbers of lung-resident ILC2s.

### IL-33-Driven *Ex Vivo* Activation of Group 2 Innate Lymphoid Cells Induces c-Rel Expression

**Figure 3:**
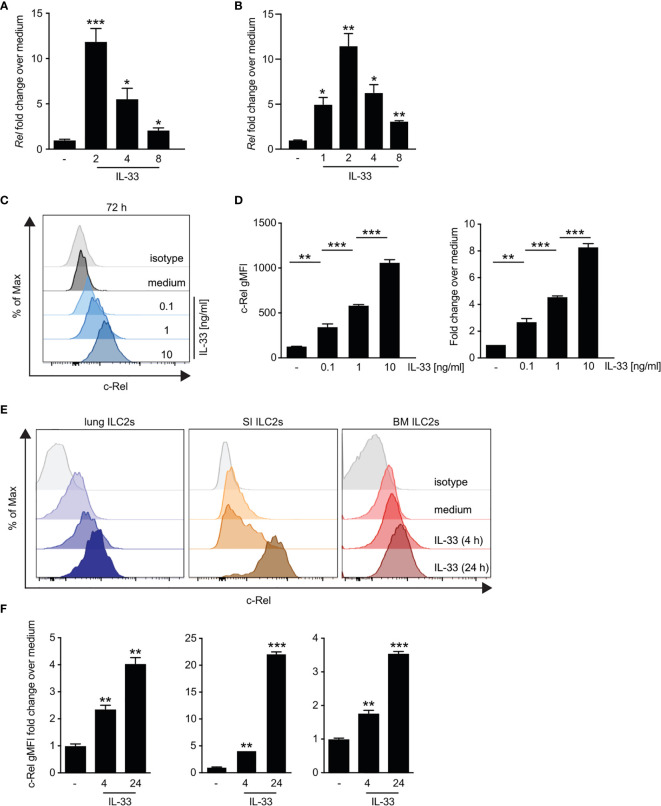
IL-33-driven *ex vivo* activation of group 2 innate lymphoid cells (ILC2s) induces c-Rel expression. **(A–D)** Murine lung, small intestine, or bone marrow-derived ILC2s were isolated by flow cytometry and left unstimulated (−) or cultured in the presence of recombinant murine IL-33 for the indicated time points. Kinetics of c-Rel (*Rel*) transcript expression in **(A)** *ex vivo* expanded bone marrow-derived ILC2s stimulated with IL-33 (10 ng/ml) or **(B)** primary murine lung ILC2s. Asterisks indicate significance over untreated control. Transcript levels were assessed by qRT-PCR and are depicted as fold change over unstimulated (−) ILC2s. Asterisks indicate significance over untreated control. **(C, D)** Flow cytometric analysis of intracellular expression of c-Rel of isolated lung ILC2s after 72 h of *ex vivo* stimulation with medium (−) or IL-33 (0.1, 1, and 10 ng/ml). c-Rel staining is shown as **(C)** histogram plots in comparison with an isotype control antibody (gray), **(D)** gMFI (left panel), or fold change over unstimulated ILC2s (right panel). Asterisks indicate significance over unstimulated control. **(E)** Representative histogram plots of intracellular flow cytometric staining of c-Rel in lung (left), small intestine (middle), or bone marrow-derived (right) ILC2s left untreated (medium) or cultured with IL-33 (10 ng/ml) for 4 or 24 h. c-Rel staining is shown in comparison with staining with an isotype control antibody (gray). **(F)** c-Rel staining intensity in lung (left), small intestinal (middle), or bone-marrow-derived (right) ILC2s quantified as gMFI fold change over unstimulated cells. Asterisks indicate significance over respective untreated controls. Data are representative of at least two independent experiments including two or more biological replicates. Data are shown as mean ± SD with *p < 0.05, **p < 0.01, and ***p < 0.001 as determined by two-tailed t-test (unpaired). gMFI, geometric mean fluorescence intensity.

Although it is well established that IL-33 signaling *via* ST2 triggers downstream activation of NF-κB pathways (8), the specific roles of the NF-κB subunit c-Rel in ILC2s remain unknown. We therefore analyzed the direct effect of IL-33 on c-Rel expression and activation in ILC2s. *Ex vivo* culture of bone marrow-derived (**Figure 3A** and **Supplementary Figure 3A**) as well as lung ILC2s (**Figure 3B** and **Supplementary Figure 2B**) with IL-33 resulted in a rapid increase in c-Rel transcripts levels compared with unstimulated cells. Consistently, lung c-Rel protein levels increased in a dose-dependent manner and remained elevated for at least 72 h of culture with IL-33 (**Figures 3C, D**). Furthermore, c-Rel protein expression in lung, small intestine, and bone marrow-derived ILC2s was significantly elevated over levels found in unstimulated cells as early as 4 h following stimulation with IL-33 and was further induced 24 h post stimulation (**Figures 3E, F** and **Supplementary Figure 3B**). Taken together, these findings show that IL-33 potently induces c-Rel expression in ILC2s derived from different anatomical locations, indicating a tissue-spanning role of the IL-33–c-Rel axis.

### c-Rel Translocates to the Nucleus Upon *Ex Vivo* Activation of Group 2 Innate Lymphoid Cells by IL-33

**Figure 4:**
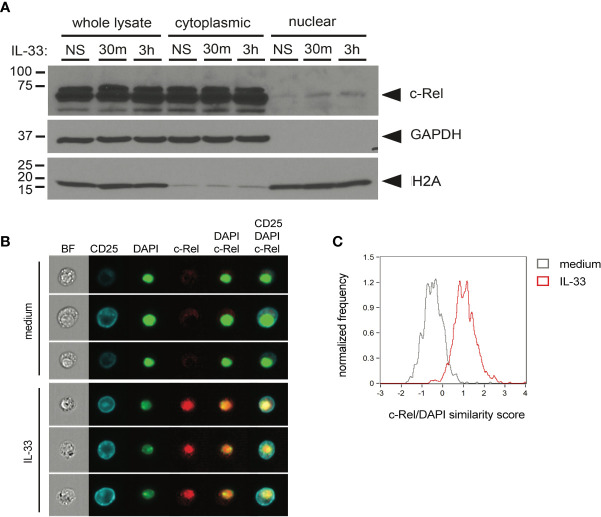
c-Rel translocates to the nucleus upon *ex vivo* activation of group 2 innate lymphoid cells (ILC2s) by IL-33. **(A)** *Ex vivo* expanded bone-marrow derived ILC2s were left unstimulated (NS) or cultured with IL-33 (100 ng/ml) for 30 min or 3 h. Whole-cell, cytoplasmic, and nuclear lysates were probed for c-Rel by Western blotting. Expression of GAPDH and H2A served as fractionation and loading controls. **(B, C)** ImageStream analysis of isolated lung ILC2s left unstimulated (medium) or cultured with IL-33 (10 ng/ml) for 3 h. **(B)** Representative images (from left to right) of bright-field (BF), CD25, DAPI, and c-Rel as well as merged images of DAPI + c-Rel and CD25 + DAPI + c-Rel. **(C)** Quantification of nuclear translocation of c-Rel by overlay of c-Rel/DAPI similarity scores of untreated (gray histogram) and IL-33-activated ILC2s (red histogram). Data are representative of at least two independent experiments.

To determine whether c-Rel is activated upon IL-33 stimulation, we assessed nuclear translocation in ILC2s upon *ex vivo* IL-33 stimulation by Western blotting using cytoplasmic and nuclear ILC2 lysates (**Figure 4A**) as well as by ImageStream analysis (**Figures 4B, C**). With both methods, we observed that c-Rel remained confined to the cytoplasm in unstimulated ILC2s, while activation with IL-33 resulted in translocation of c-Rel from the cytoplasm to the nucleus, indicating a potential role of c-Rel in transcriptional regulation of ILC2 effector functions.

### c-Rel Expression Is Induced in Lung Group 2 Innate Lymphoid Cells Following Intranasal Challenge With IL-33

**Figure 5:**
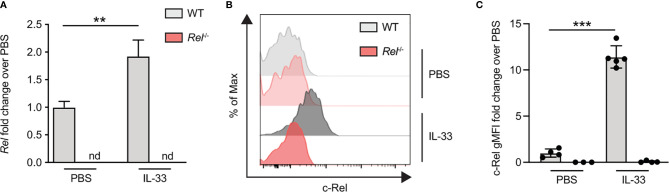
c-Rel expression is induced in lung group 2 innate lymphoid cells (ILC2s) following intranasal challenge with IL-33. Wild-type (WT) (gray) and *Rel* ^−/−^ (red) mice were intranasally challenged for three consecutive days (days 0, 1, and 2) with either phosphate-buffered saline (PBS) or IL-33 (500 ng/mouse), and lungs were analyzed 24 h after the last administration. **(A)** Lung c-Rel transcript levels in WT and *Rel^−/−^* mice following intranasal challenge were determined by qRT-PCR, and **(B, C)** intracellular expression of c-Rel protein in lung ILC2s was analyzed by flow cytometry. **(B)** Representative histogram plots of intracellular c-Rel staining in pulmonary WT and *Rel* ^−/−^ ILC2s following intranasal challenge with PBS or IL-33 quantified as **(C)** gMFI fold change over the PBS-treated control group. Data points are representative of at least two independent experiments with n = 3–5 mice per treatment group. Data are shown as mean ± SD with **p < 0.01, and ***p < 0.001 as determined by one-way ANOVA followed by Tukey’s multiple comparisons test or by two-tailed t-test (unpaired). gMFI, geometric mean fluorescence intensity; nd, not detectable.

To assess if c-Rel expression is also increased upon *in vivo* activation of ILC2s, we induced allergic airway inflammation by treating mice intranasally with IL-33 for 3 days. c-Rel-deficient mice that underwent the same treatment were used as negative controls. c-Rel transcript levels were significantly higher in lung tissues of IL-33-challenged mice compared with mice that received PBS, while no transcripts were detected in the lungs of control *Rel* ^−/−^ mice (**Figure 5A**). Consistent with ILC2s stimulated *ex vivo*, intranasal administration of IL-33 in WT mice resulted in a 10-fold induction of c-Rel expression in lung ILC2s over PBS-treated mice (**Figures 5B, C**). Taken together, these findings show that c-Rel expression is induced upon *ex vivo* stimulation of ILC2s as well as during IL-33- and allergen-induced allergic airway inflammation.

### c-Rel Deficiency Limits Group 2 Innate Lymphoid Cell Effector Functions Following *Ex Vivo* Activation

**Figure 6:**
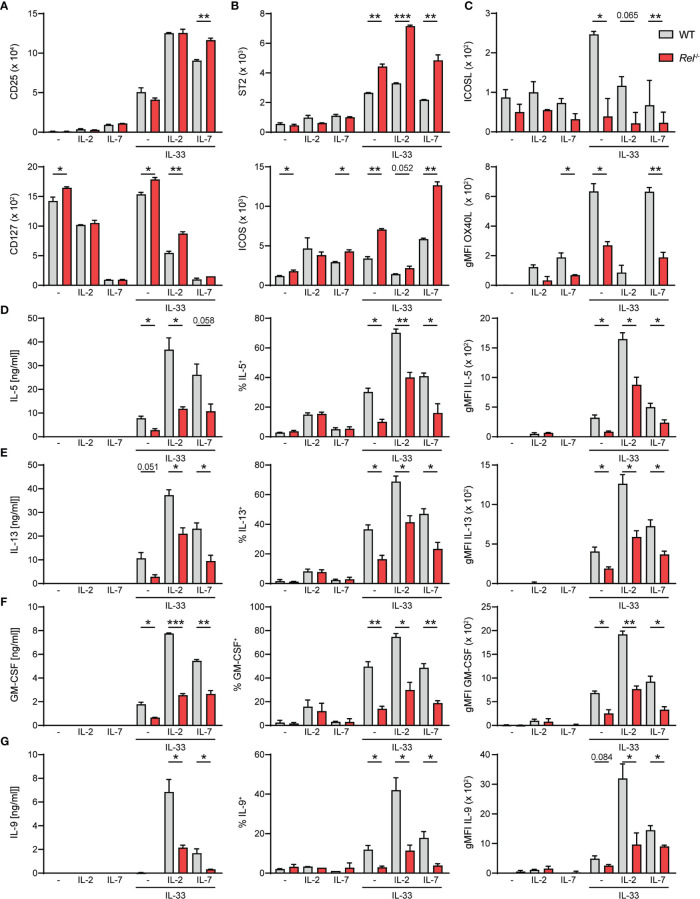
c-Rel deficiency limits group 2 innate lymphoid cell (ILC2) effector functions following *ex vivo* activation. Lung ILC2s from wild-type (WT) and *Rel* ^−/−^ mice were isolated by flow cytometry and cultured *ex vivo* in the absence (−) or presence of IL-2, IL-7, or IL-33 and combinations thereof (all 10 ng/ml). Expression levels of surface **(A)** CD25 and CD127, **(B)** ST2 and ICOS, and **(C)** ICOSL and OX40L on isolated WT (gray) and *Rel* ^−/−^ (red) lung ILC2s after 24-h stimulation under indicated conditions. Analysis of **(D)** IL-5, **(E)** IL-13, **(F)** granulocyte-macrophage colony-stimulating factor (GM-CSF), and **(G)** IL-9 expression by WT (gray) and *Rel* ^−/−^ (red) lung ILC2s left untreated or cultured with IL-33 for 48 h. Cytokine concentration in culture supernatants is depicted in the left panel, frequencies of cytokine-producing cells are depicted in the middle panel, and cytokine expression levels are depicted as gMFI in the right panel. Data points are representative of two independent experiments with two biological replicates for each stimulation condition. Data are shown as mean ± SD with *p < 0.05, **p < 0.01, and ***p < 0.001 as determined by two-tailed t-test (unpaired). gMFI, geometric mean fluorescence intensity.

Besides activating signals provided by alarmins, ILC2s require cues from STAT5-activating cytokines such as IL-2, IL-7, or TSLP, as well as costimulatory signals for optimal activation and exertion of effector functions (6, 26). We therefore assessed whether observed reduction in *Rel* ^−/−^ ILC2 numbers during allergic airway inflammation may stem from lowered sensitivity to activating signals due to diminished expression of the respective surface receptors. To this end, we assessed surface expression levels of CD25 (IL-2Rα), CD127 (IL-7R), and other key activating ILC2 receptors as well as the ILC2 master transcription factor GATA3 in isolated pulmonary WT and *Rel* ^−/−^ ILC2s at steady state and after *ex vivo* stimulation with IL-33 in the presence or absence of IL-2 or IL-7. We found that c-Rel deficiency did not result in diminished expression of surface CD25, CD127, or GATA3 by ILC2s at steady state or upon IL-33-mediated activation under the tested conditions (**Figures 6A** and **Supplementary Figure 4A**). Moreover, the IL-33R chain ST2 and the costimulatory receptor ICOS (**Figure 6B**) were slightly enhanced upon ILC2 activation in c-Rel-deficient ILC2s when compared with WT cells.

Besides ICOS, murine ILC2s also express ICOS ligand (ICOSL), whose interaction with ICOS promotes ILC2 survival and effector cytokine production upon IL-33-mediated activation (27, 28). Furthermore, constitutive as well as activation-induced expression of other costimulatory ligands and receptors including DR3, GITR, and OX40L on ILC2s have been reported (13, 29–32). We therefore assessed whether c-Rel regulates the expression of costimulatory ligands and/or receptors that promote ILC2 functions directly as well as are expressed by ILC2s to aid in instructing adaptive immune responses. While no changes in DR3 or GITR expression were observed (data not shown), deficiency in c-Rel resulted in significantly reduced expression of surface ICOSL as well as OX40L (**Figure 6C**), indicating that c-Rel might further enhance ILC2 functions by driving ICOSL expression and also potentially drive adaptive type 2 responses by positively regulating surface OX40L levels.

Earlier studies demonstrated that c-Rel promotes cell cycle progression and survival in activated lymphocytes (21, 33–36). We therefore analyzed the proliferative capacity of lung ILC2s at steady state or upon IL-33-driven activation by Ki-67 staining after 48 h of activation. Ki-67 expression levels in lung ILC2s isolated from WT and *Rel* ^−/−^ mice were comparable upon IL-33 stimulation, indicating that c-Rel does not impact early expansion of IL-33-activated ILC2s (**Supplementary Figure 4B**).

ILC2s mediate their effector mechanisms by rapidly secreting large amounts of type 2 signature cytokines upon activation. To determine whether c-Rel directly regulates lung ILC2 type 2 signature cytokine production, we determined the concentrations of IL-5, IL-13, GM-CSF, and IL-9 in culture supernatants of ILC2s that were left unstimulated or were cultured with IL-2, IL-7, or IL-33 alone, or with indicated combinations (**Figures 6D–G**). We additionally assessed frequencies of cytokine-producing ILC2 and intracellular cytokine expression levels. All measured type 2 cytokines were induced upon activation of ILC2s with IL-33, and expression was further elevated when ILC2s were stimulated with IL-33 together with IL-2 or IL-7. Importantly, effector cytokine levels in the culture supernatant were significantly reduced in the absence of c-Rel (**Figures 6D–G**, left panel), which may be a direct result of significantly lower frequencies of cytokine-producing ILC2s (**Figures 6D–G**, middle panel) as well as significantly diminished production of cytokines within those cells (**Figures 6D–G**, right panel). Collectively, our data show that c-Rel positively regulates the expression of costimulatory ligands as well as ILC2 effector cytokines, thereby driving innate and potentially also adaptive type 2 immune responses.

### c-Rel Drives Type 2 Effector Responses During IL-33-Induced Allergic Airway Inflammation

**Figure 7:**
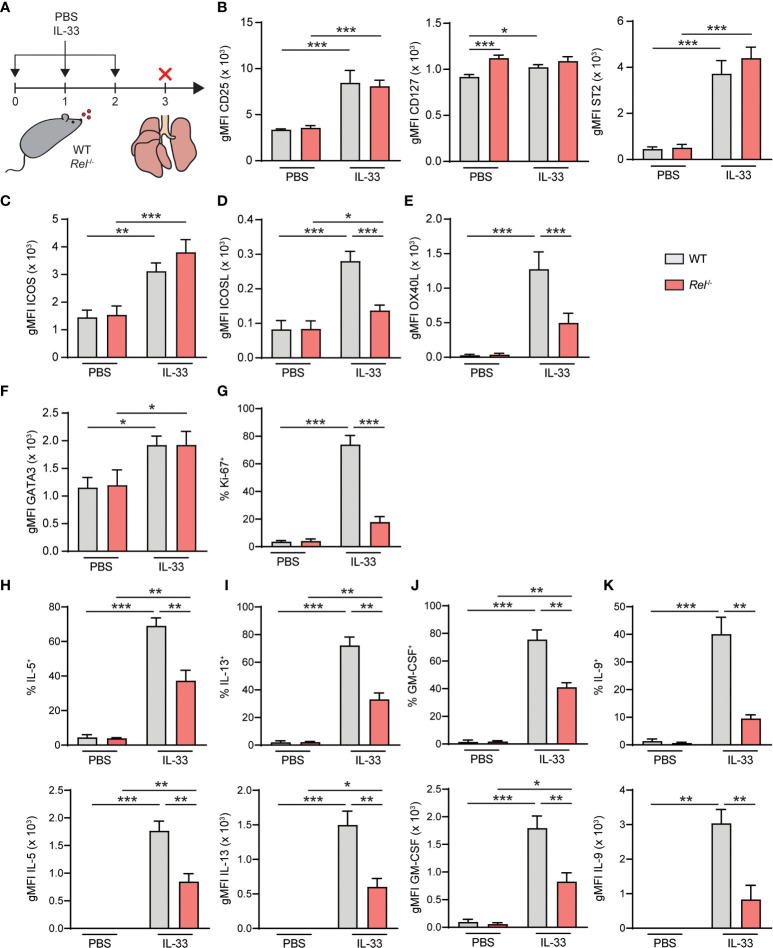
c-Rel drives group 2 innate lymphoid cell (ILC2) effector responses during IL-33-induced allergic airway inflammation. **(A)** Wild-type (WT) and *Rel* ^−/−^ mice were challenged intranasally for 3 days (days 0, 1, and 2) with phosphate-buffered saline (PBS) or IL-33 (500 ng/mouse). Mice were sacrificed, and lungs were analyzed 24 h after the last administration. Flow cytometry analysis of surface expression levels of **(B)** CD25, CD127, and ST2, **(C)** ICOS, **(D)** ICOSL, and **(E)** OX40L, and intracellular expression levels of **(F)** GATA3 in lung ILC2s following intranasal challenge of WT (gray bars) and *Rel* ^−/−^ (red bars) mice. **(G)** Frequencies of Ki-67^+^ WT (gray bars) and *Rel* ^−/−^ (red bars) lung ILC2s following *in vivo* challenge. Analysis of intracellular expression of **(H)** IL-5, **(I)** IL-13, **(J)** granulocyte-macrophage colony-stimulating factor (GM-CSF), and **(K)** IL-9 in WT (gray bars) and *Rel* ^−/−^ (red bars) in lung ILC2s following intranasal PBS or IL-33 challenge and *ex vivo* restimulation with phorbol myristate acetate (PMA)/ionomycin. Frequencies of cytokine-expressing cells are depicted in the top panel; and expression levels, measured as gMFI, are shown in the bottom panel. Data points are representative of two independent experiments with two biological replicates for each stimulation condition. Data are shown as mean ± SD with *p < 0.05, **p < 0.01, and ***p < 0.001 as determined by two-tailed t-test (unpaired). gMFI, geometric mean fluorescence intensity.

To further decipher the *in vivo* impact of c-Rel on ILC2 function, WT and *Rel* ^−/−^ mice were challenged intranasally for three consecutive days with PBS as a control or IL-33 (**Figure 7A**). Mice were sacrificed and lung ILC2 surface expression levels of CD25, CD127, and ST2 (**Figure 7B**), ICOS as well as ICOSL and OX40L were determined. Additionally, we assessed intracellular expression levels of GATA3 (**Figure 7F**) and frequencies of Ki-67^+^ proliferating lung ILC2s following *in vivo* challenge. In accordance with our *ex vivo* stimulation results, no significant differences were observed in the expression of the key stimulatory ILC2 receptors CD25, CD127, and ST2 and ICOS (**Figures 7B, C**). Importantly, both ICOSL and OX40L were significantly downregulated in the absence of c-Rel, suggesting an *in vivo* role of c-Rel in the regulation of ILC2 costimulation (**Figures 7D, E**). While GATA3 expression was comparable in ILC2s of WT and c-Rel-deficient mice (**Figure 7F**), frequencies of proliferating ILC2s were significantly reduced in the absence of c-Rel (**Figure 7G**).

To determine the effect of c-Rel on ILC2 effector cytokine production, lung cells from challenged animals were activated with phorbol myristate acetate (PMA)/ionomycin; and intracellular levels of IL-5 (**Figure 7H**), IL-13 (**Figure 7I**), GM-CSF (**Figure 7J**), and IL-9 (**Figure 7K**) in ILC2s were analyzed. While production of all cytokines was induced, frequencies of cytokine-expressing ILC2s were significantly reduced in *Rel* ^−/−^ mice following *in vivo* challenge (**Figures 7H–K**, top panel). Furthermore, intracellular cytokine expression levels, measured as gMFI, were markedly lower in the absence of c-Rel. Together, these data show that c-Rel is critical for ILC2 proliferation as well as type 2 effector cytokine production during allergic airway inflammation and might potentially drive subsequent adaptive immune responses by promoting the expression of OX40L.

## Discussion

ILC2s are critical mediators of early type 2 immune responses and allergic airway disease. They depend on an intricate network of transcriptional regulators to ensure efficient activation and execution of effector functions. Rel/NF-κB transcription factors modulate immune responses by regulating the expression of hundreds of genes involved in lymphoid cell development, proliferation, survival, and immune cell effector functions (37).

We here observed that the NF-κB transcription factor c-Rel promotes type 2 immunity in an ILC2-driven mouse model of allergic airway inflammation. Challenge of c-Rel-deficient mice resulted in reduced lung immune cell infiltration and diminished numbers of key type 2 immune cell populations including ILC2s, cDC2s, and eosinophils. Consistent with reduced ILC2 numbers, markedly lower transcript and protein levels of the type 2 signature cytokine IL-5 were observed, which is critical for the recruitment of eosinophils to the lung during allergic airway inflammation (38). Moreover, c-Rel deficiency led to lower pulmonary IL-13 transcript and protein levels, a hallmark cytokine of type 2 immunity. Attenuated induction of the type 2 chemokine ligands CCL17 and CCL22 in the lungs of *Rel^−/−^* mice following exposure to IL-33 was observed as well, which may be a direct result of reduced numbers of CCL17- and CCL22-producing DC2s (39). Moreover, CCL17 and CCL22 have been described previously to promote ILC2 chemotaxis, and reduced levels might affect positioning of ILC2s within the airways, which could contribute to the observed reduction of type 2 responses (40). In addition, we demonstrate that c-Rel deficiency in ILC2s leads to markedly reduced IL-9 and GM-CSF production *in vitro* and *in vivo*. IL-9 has been shown to increase ILC2 fitness (41) and reinforces innate and adaptive type 2 immune responses (42, 43). In addition, GM-CSF has been shown to drive type 2 immunity (44) and allergic sensitization (45). This suggests that the attenuated response of c-Rel-deficient mice to IL-33 may be the result of defective activation and cytokine production by ILC2s.

Consistent with earlier reports showing that c-Rel is dispensable for normal hemopoiesis and lymphocyte development, the absence of c-Rel did not result in altered numbers of ILC2 precursors (21). In addition, numbers of lung ILC2s were comparable in naive WT and *Rel^−/−^* mice, indicating no homeostatic defects due to c-Rel deficiency that might contribute to the inability of c-Rel-deficient mice to mount an efficient type 2 response upon IL-33 challenge.

Our data show that c-Rel expression is potently induced in a dose-dependent manner in pulmonary ILC2s at both transcript and protein levels following activation with IL-33. Upregulation of c-Rel protein levels was also observed in IL-33-stimulated ILC2s isolated from other tissues, indicating a general role of c-Rel in ILC2 function. Importantly, c-Rel expression was induced in lung ILC2s following intranasal challenge with IL-33, suggesting a physiological function during allergic airway inflammation. We furthermore demonstrated nuclear translocation of c-Rel in lung ILC2s upon IL-33 stimulation, and since c-Rel harbors a transcription transactivation domain, it can thereby act as a direct transcriptional activator once in the nucleus (37).

Our data suggest that the attenuated response of c-Rel-deficient mice to IL-33 may be the result of defective activation and expansion of ILC2s. IL-33 acts in concert with signals provided by costimulatory STAT5-activating cytokines IL-2 and IL-7, and costimulatory surface molecules to promote ILC2 survival, expansion, and exertion of effector functions (6, 26). Previous work demonstrated that c-Rel upregulates the expression of IL-2Rα (CD25) in T cells (21, 33, 46, 47), and it is known that IL-2 enhances ILC2 survival as well as activation-mediated expansion and type 2 cytokine production (48–50). Moreover, earlier studies demonstrated that c-Rel positively modulates cell cycle progression and the expression of anti-apoptotic proteins in activated T and B cells (21, 33–36). In addition, genes encoding the type 2 signature cytokines IL-4 and IL-13 were proposed as putative c-Rel target genes based on differential transcript expression observed in gain-of-function and loss-of-function experiments in T cells (51). While our work showed no effect of c-Rel on ILC2-intrinsic expression of the cytokine receptors CD25 and CD127, we observed a strong reduction of IL-5, IL-9, IL-13, and GM-CSF production upon IL-33 stimulation *in vitro* and *in vivo*. Similar to T cells, ILC2s express activating costimulatory molecules, including DR3, GITR, and ICOS, that promote ILC2 function in disease settings as well as during homeostasis (13, 29–31, 52, 53). While no reduction in ICOS expression was observed, ICOSL and OX40L expression was found to be significantly lower in c-Rel deficient ILC2 after activation. This could explain the markedly reduced ILC2 response in the absence of c-Rel during experimental acute allergic airway inflammation.

Interestingly, WT and c-Rel deficient ILC2s proliferated equally well *in vitro*. In contrast, the proliferative potential was found to be altered *in vivo*. Since c-Rel is expressed in a variety of cells within the hematopoietic compartment (10, 54), it cannot be excluded that c-Rel deficiency impacts effector functions of other innate as well as adaptive pulmonary immune cell populations such as IL-33-reactive macrophages, DCs, or CD4^+^ T helper cells, which could potentially promote ILC2 activation by providing costimulation and/or activating cytokines. IL-33 was shown to induce IL-2 expression in DCs (55) and IL-2 is known to strongly enhance ILC2 survival as well as activation-mediated expansion and type 2 cytokine production (48–50). Moreover, interaction of ILC2 with CD4^+^ T cells has been suggested to enhance the ability of CD4^+^ T cells to produce IL-2, thereby reinforcing ILC2 activation (49). Interestingly, c-Rel deficiency was shown to alter the ability of CD4^+^ T cells to produce IL-2 (46, 56). It is therefore possible that c-Rel deficiency also alters the capacity of CD4^+^ T cells to produce IL-2 in response to IL-33, which could contribute to the altered proliferative capacity of ILC2 observed *in vivo*.

Our data using *Rel* ^−/−^ mice suggest that c-Rel promotes acute ILC2-driven allergic airway inflammation and suggests that c-Rel may contribute to the pathophysiology of ILC2-mediated allergic airway disease. It thereby represents a promising target for the treatment of allergic asthma, and evaluating the effect of established c-Rel inhibitors in this context would be of great clinical interest. In addition, while RelA deficiency results in embryonic lethality and *RelA* ^−/−^ cells exhibit severe defects regarding survival, proliferation, and effector functions (57), mice lacking c-Rel are viable and only show limited immunological defects (21). Thus, targeting c-Rel specifically may avoid the adverse side effects that have halted advancement of pan-NF-κB inhibitors for therapeutic applications (19, 58). IL-33-dependent ILC2 activation has also been reported during cancer (59, 60), viral infections (22, 61, 62), and murine models of autoimmune diseases (63–66); and given the observed induction of c-Rel expression in ILC2s from different tissues, modulation of c-Rel function in these conditions may be worth evaluating.

## Data Availability Statement

The datasets presented in this article are not readily available because no datasets were generated as listed in 2. Requests to access the datasets should be directed to jorg.fritz@mcgill.ca.

## Ethics Statement

The animal study was reviewed and approved by the Canadian Council on Animal Care, McGill University, ethics committee.

## Author Contributions

JF and BM designed the intellectual framework of the study and the layout of the manuscript. BM, SK, and CD performed *in vivo* allergic airway inflammation models, and BM and SK conducted *ex vivo* cell culture experiments. BM, SK, CD, and DL analyzed data. MM carried out cellular fractionation and Western blotting analysis. LR performed histological scoring. SV and SG provided critical reagents and mouse strains. BM and JF designed experiments and wrote the manuscript. All authors contributed to the article and approved the submitted version.

## Funding

The work in the laboratory of JF is supported by a foundation (FDN-148405) followed by a project grant (PJT-175173) from the Canadian Institutes of Health Research (CIHR), and a Leaders Opportunity Fund infrastructure grant from the Canadian Foundation of Innovation (CFI; #38958). JF is supported by a CIHR New Investigator Award and by a Junior 1 & 2 Investigator Award by the (FRQS). DL’s work is supported by a Canada Foundation for Innovation John R. Evans Leaders Fund award (CFI-JELF #38033), Startup Funds from the McGill University Faculty of Medicine, and a CIHR Project Grant (#168959). DL is also recipient of a FRQS, Chercheur-Boursier Junior 1 Award (#284497). CD acknowledges support by a Rahel Hirsch stipend.

## Conflict of Interest

The authors declare that the research was conducted in the absence of any commercial or financial relationships that could be construed as a potential conflict of interest.

## Publisher’s Note

All claims expressed in this article are solely those of the authors and do not necessarily represent those of their affiliated organizations, or those of the publisher, the editors and the reviewers. Any product that may be evaluated in this article, or claim that may be made by its manufacturer, is not guaranteed or endorsed by the publisher.

## Supplementary Material

Supplementary Figure 1 Gating strategies for the identification of pulmonary type 2 cell populations. **(A)** Multicolor flow cytometry gating strategies to identify murine pulmonary **(B)** eosinophils (single, live CD45^+^SSC^hi^Siglec-F^+^CD11c^-^), **(C)** cDC2s (single, live CD45^+^CD64^-^ Lin^-^MHCII^+^CD26^+^CD11c^+^XCR1-CD172a^+^) as well as **(D)** ILC2s (single, live CD45^+^ Lin^-^Thy-1^+^CD127^+^KLRG1^+^). cDC2, conventional type 2 dendritic cell.

Supplementary Figure 2 Gating strategies for the identification of bone marrow ILC2 progenitor populations and isolation of murine lung ILC2s. Flow cytometry gating strategies to **(A)** identify bone marrow CLP (single live Lin^-^CD127^+^Flt3^+^a4b7^-^), CHILP (single live Lin^-^CD127^+^Flt3^+^a_4_b_7_^+^CD25^-^) and ILC2P (single live Lin-CD127^+^Flt3^+^a_4_b_7_^+^ CD25^+^) populations and **(B)** isolate murine lung ILC2s (single live Lin-CD45^+^Thy-1^+^ST2^+^CD25^+^). CLP, common lymphoid progenitor; CHILP, common helper ILC2 progenitor; ILC2P, ILC2 progenitor.

Supplementary Figure 3 Gating strategies for the isolation of murine bone marrow-derived ILC2 progenitors and small intestinal ILC2s. Flow cytometry gating strategies to isolate **(A)** bone marrow-derived ILC2 progenitors (single Lin^-^ Sca-1^+^c-Kit^-^ CD25^+^) and **(B)** murine small intestinal ILC2s (single live CD45^+^Lin^-^ KLRG1^+^CD127^+^).

Supplementary Figure 4 Absence of c-Rel does not affect GATA3 expression or early ILC2 proliferation upon *ex vivo* IL-33 challenge. Isolated WT (grey bars) and *Rel* ^-/-^ (red bars) lung ILC2s were left untreated (-) or cultured with IL-2, IL-7, IL-33 (all 10 ng/ml) or indicated combinations for 48 hours and analyzed by flow cytometry. **(A)** GATA3 expression, shown as gMFI. **(B)** Frequencies of Ki-67-expressing lung ILC2s. Data points are representative of two independent experiments with two biological replicates for each stimulation condition. Data are shown as mean ± SD with **p < 0.01 as determined by two-tailed t test (unpaired). gMFI, geometric mean fluorescence intensity.
