# Supplementary material for: The NF-κB Transcription Factor c-Rel Modulates Group 2 Innate Lymphoid Cell Effector Functions and Drives Allergic Airway Inflammation
Source: Front Immunol. 2021 Nov 16;12:664218. doi: 10.3389/fimmu.2021.664218 (PMC8635195; doi:10.3389/fimmu.2021.664218)
Supplement: Supplementary Figure 1 — Gating strategies for the identification of pulmonary type 2 cell populations. (A) Multicolor flow cytometry gating strategies to identify murine pulmonary (B) eosinophils (single, live CD45+SSChiSiglec-F+CD11c-), (C) cDC2s (single, live CD45+CD64- Lin-MHCII+CD26+CD11c+XCR1-CD172a+) as well as (D) ILC2s (single, live CD45+ Lin-Thy-1+CD127+KLRG1+). cDC2, conventional type 2 dendritic cell. [file Image_1.pdf]

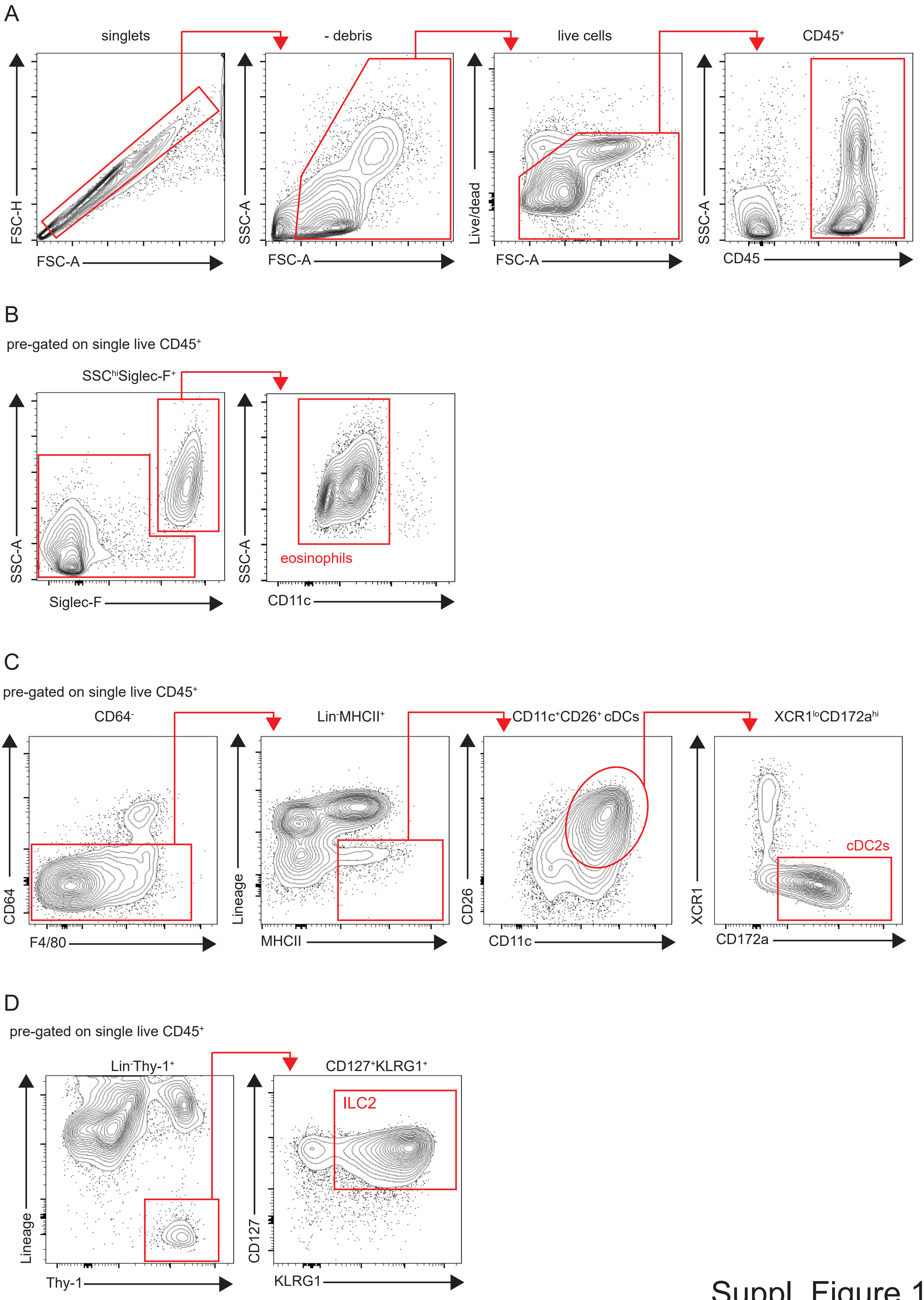


Suppl. Figure 1
