# Supplementary figures and images for: The NF-κB Transcription Factor c-Rel Modulates Group 2 Innate Lymphoid Cell Effector Functions and Drives Allergic Airway Inflammation
Source: Front Immunol. 2021 Nov 16;12:664218. doi: 10.3389/fimmu.2021.664218 (PMC8635195; doi:10.3389/fimmu.2021.664218)

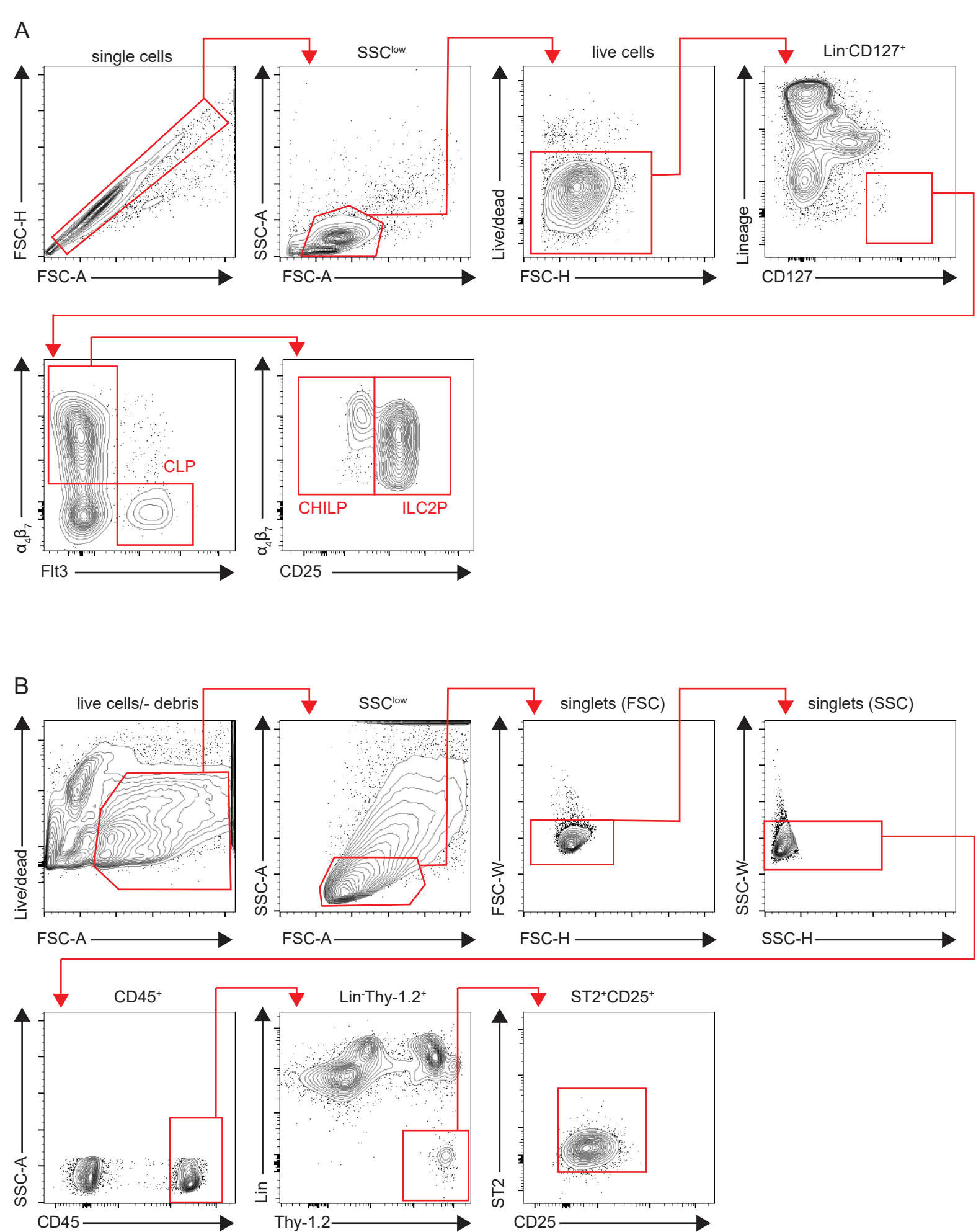

Supplement: Supplementary Figure 2 — Gating strategies for the identification of bone marrow ILC2 progenitor populations and isolation of murine lung ILC2s. Flow cytometry gating strategies to (A) identify bone marrow CLP (single live Lin-CD127+Flt3+a4b7-), CHILP (single live Lin-CD127+Flt3+a4b7 +CD25-) and ILC2P (single live Lin-CD127+Flt3+a4b7 + CD25+) populations and (B) isolate murine lung ILC2s (single live Lin-CD45+Thy-1+ST2+CD25+). CLP, common lymphoid progenitor; CHILP, common helper ILC2 progenitor; ILC2P, ILC2 progenitor. [file Image_2.pdf]

A

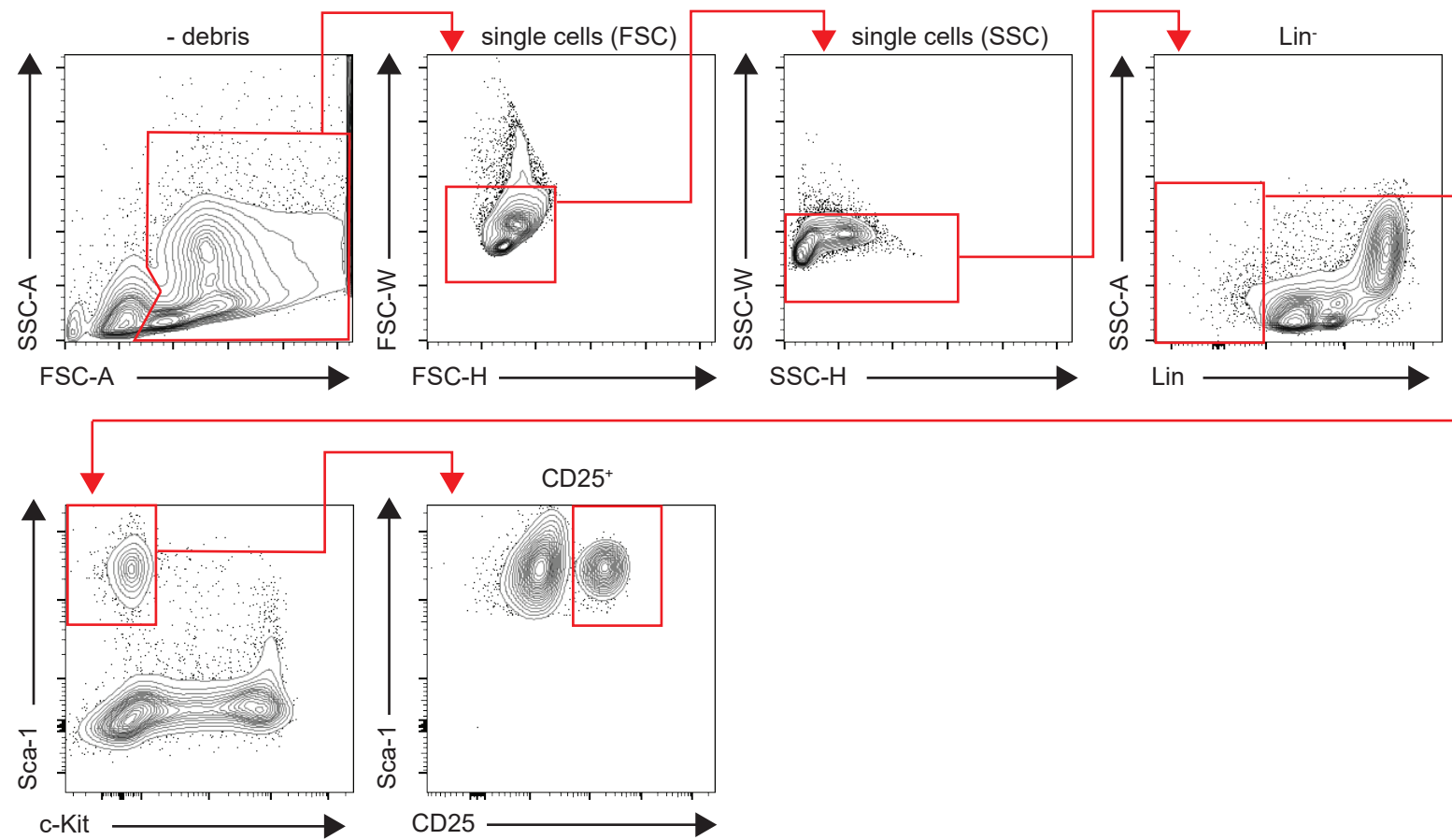

B

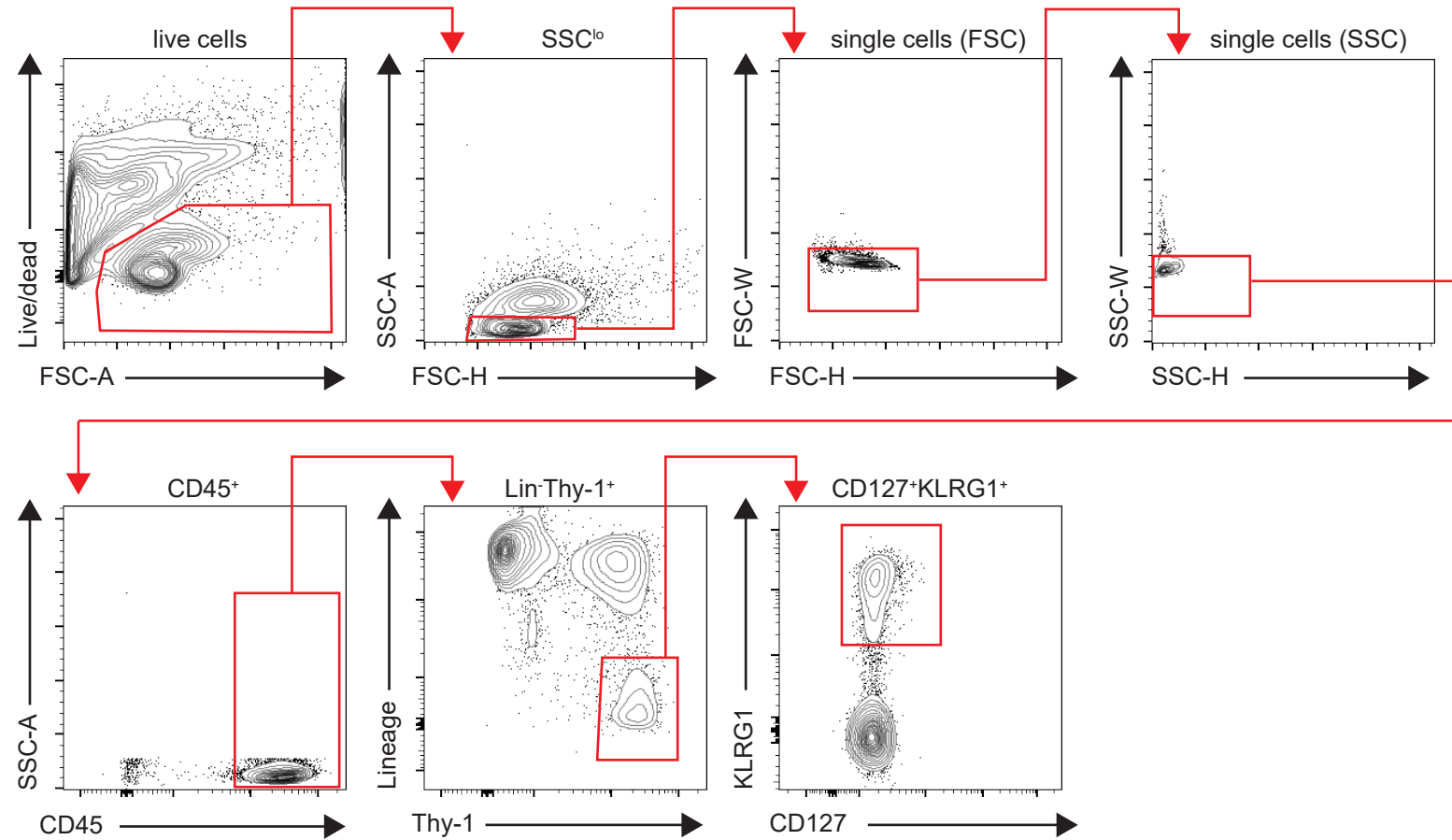

Supplement: Supplementary Figure 3 — Gating strategies for the isolation of murine bone marrow-derived ILC2 progenitors and small intestinal ILC2s. Flow cytometry gating strategies to isolate (A) bone marrow-derived ILC2 progenitors (single Lin- Sca-1+c-Kit- CD25+) and (B) murine small intestinal ILC2s (single live CD45+Lin- KLRG1+CD127+). [file Image_3.pdf]

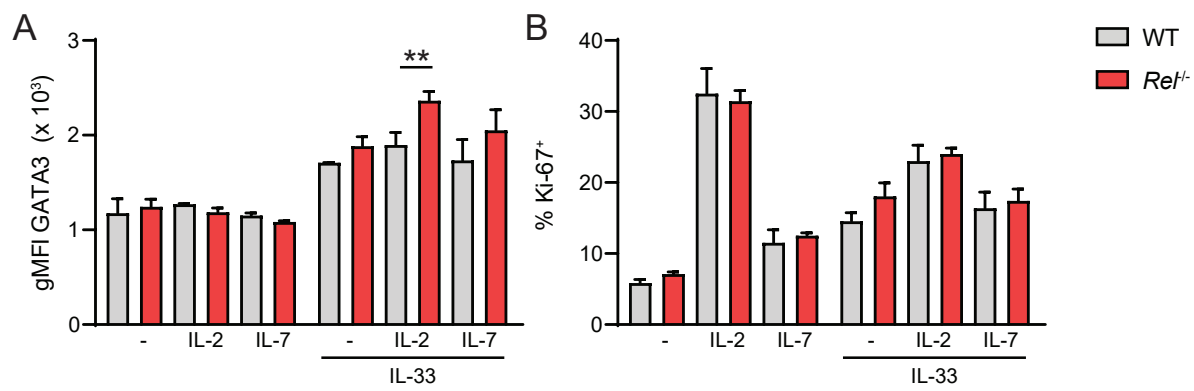

Supplement: Supplementary Figure 4 — Absence of c-Rel does not affect GATA3 expression or early ILC2 proliferation upon ex vivo IL-33 challenge. Isolated WT (grey bars) and Rel -/- (red bars) lung ILC2s were left untreated (-) or cultured with IL-2, IL-7, IL-33 (all 10 ng/ml) or indicated combinations for 48 hours and analyzed by flow cytometry. (A) GATA3 expression, shown as gMFI. (B) Frequencies of Ki-67-expressing lung ILC2s. Data points are representative of two independent experiments with two biological replicates for each stimulation condition. Data are shown as mean ± SD with **p < 0.01 as determined by two-tailed t test (unpaired). gMFI, geometric mean fluorescence intensity. [file Image_4.pdf]
